# Apathy in the Elderly: From Assessment to Treatment

**DOI:** 10.1155/2012/419309

**Published:** 2012-09-13

**Authors:** Iracema Leroi, Philippe H. Robert

**Affiliations:** ^1^Department of Psychiatry and Behavioural Sciences, School of Community Based Medicine, University of Manchester, Manchester, UK; ^2^Centre Mémoire de Ressources et de Recherche, CHU University of Sophia Antipolis, Nice, France

In a clinical context, the term “apathy” refers to a clustering of behavioural and emotional symptoms that manifests as diminished interest and involvement in normal purposeful behaviour, diminished emotional responsiveness, and a lack of initiation and drives to complete nonroutine activities. Apathy may be one of the most common behavioural symptoms of neurodegenerative and other brain disorders yet it is one of the most underrecognised, underdiagnosed, and poorly managed aspects of these diseases. It is therefore gratifying that we have been able to produce a special issue on the topic of apathy covering aspects of significant interest to the practicing clinician. 

The papers in this issue encompass a broad spectrum of clinical aspects of apathy with a clear overarching theme of apathy as an important behavioural syndrome which warrants careful study of its features, diagnosis and management. The specific topics represented by the papers range from an exploration of the phenomenology of apathy and the manifestation of apathy in different neurodegenerative conditions, the overlap between apathy and depression, and the application of new technologies in the management of apathy in the nursing home settings. 

Behavioural syndromes such as apathy may inform diagnostic aspects of different types of dementia. In the paper by D. Quaranta et al., apathy-related items on the Neuropsychiatric Inventory [1] were compared in frontotemporal dementia and Alzheimer disease. Their findings suggest that the different anatomical substrates of the respective dementia types may drive the different clinical expression of apathy, which may, in turn, inform the diagnosis of the dementia type.

The link between apathy and depression as well as apathy and impulsivity was explored in two contributions. D. Ahearn et al. found that in Parkinson's disease, apathy and impulsivity might overlap and that specific phenomenological profiles within the behavioural syndromes were associated with particular clinical phenotypes. P. Njomboro and S. Deb found that in a sample of patients with acquired brain injury, the degree of overlap between depression and apathy was dependent on the method of assessment of apathy. The assessment and measurement of apathy continue to be refined as new instruments to be developed and validated. Here, item response theory [2] was used as a basis to explore a possible early screening tool for apathy in elderly people.

The management of apathy has long been a challenge and the evidence base for pharmacological interventions is very limited. It is therefore heartening to read that other approaches have been adopted. The paper by A. Leone et al. outlines a nonpharmacological intervention incorporating the use of novel technologies in the assessment and delivery of a tailored activity in nursing home residents. Further studies of interventions along these lines would be welcomed by the field.

Finally, this set of five informative papers may well become a standard feature in the reference lists of future studies in what we can now see as a pivotal topic in the overall understanding of dementia.

*Iracema Leroi* *Philippe H. Robert*
